# HIV Prevention Messages Targeting Young Latino Immigrant MSM

**DOI:** 10.1155/2014/353092

**Published:** 2014-04-17

**Authors:** Rosa Solorio, Pamela Norton-Shelpuk, Mark Forehand, Marcos Martinez, Joel Aguirre

**Affiliations:** ^1^Department of Health Services, School of Public Health, University of Washington, 4333 Brooklyn Avenue NE, Box 359455, Seattle, WA 98195, USA; ^2^Department of Global Health, School of Public Health, University of Washington, Seattle, WA 98104, USA; ^3^Social and Behavioral Prevention Core, Center for AIDS Research, University of Washington, Seattle, WA 98104-2499, USA; ^4^Activate Brands, Denver, CO 80202, USA; ^5^Foster School of Business, University of Washington, Seattle, WA 98105, USA; ^6^Entre Hermanos, Seattle, WA 98122, USA

## Abstract

Young Latino immigrant men who have sex with men (MSM) are at risk for HIV and for delayed diagnosis. A need exists to raise awareness about HIV prevention in this population, including the benefits of timely HIV testing. This project was developed through collaboration between University of WA researchers and Entre Hermanos, a community-based organization serving Latinos. Building from a community-based participatory research approach, the researchers developed a campaign that was executed by Activate Brands, based in Denver, Colorado. The authors (a) describe the development of HIV prevention messages through the integration of previously collected formative data; (b) describe the process of translating these messages into PSAs, including the application of a marketing strategy; (c) describe testing the PSAs within the Latino MSM community; and (c) determine a set of important factors to consider when developing HIV prevention messages for young Latino MSM who do not identify as gay.

## 1. Introduction

In the United States, Latinos are disproportionately affected by HIV infection and have an HIV diagnosis rate that is three times that of non-Latino Whites [1]. Late diagnosis is a significant public health problem among Latino immigrants in the United States. In the USA, Latinos are more likely than other racial/ethnic groups to receive an AIDS diagnosis within one year of testing positive for HIV [2]. Given that unrecognized HIV infections are one of the driving forces of ongoing HIV transmission among populations [3, 4], increasing screening is of upmost importance. Additionally, individuals who are infected with HIV but are unaware of their status may be more infectious than those receiving treatment because of high viral loads [5–7]. It has been estimated that unrecognized infections account for as much as 50% of new infections among general populations of MSM in the USA [4]. To bring HIV transmission under control, universal voluntary HIV testing and access to treatment have been proposed as important strategies.

Improvements in late HIV diagnoses have been achieved among USA-born populations, in the past decade [8]. However, there has been no corresponding change among immigrant populations. In places like Washington State, over 70% of Latino immigrants continue to be diagnosed late [8]. Previous studies report that Latino immigrants with low levels of English proficiency who have resided in the USA for less than five years are at an elevated risk for delayed HIV diagnosis [9, 10]. It is therefore essential for Spanish language interventions to be developed to address the HIV prevention needs of the Spanish speaking population. The Affordable Care Act will allow immigrants who reside in the USA legally to participate in the health insurance exchange programs and thus immigrant men, especially MSM, need to be made aware of the benefits of timely HIV testing; in addition, several states provide access to HIV treatment for undocumented immigrants through the Ryan White HIV/AIDS Program.

A need for research exists to identify culturally specific ways to communicate with Latino immigrant MSM and address their HIV prevention needs, including the use of social media marketing to more effectively communicate the benefits from timely HIV testing. Social media marketing is the systematic application of commercial marketing concepts and techniques to the analysis, planning, execution, and evaluation of programs and has been recognized as a way to influence the voluntary behavior of audiences to improve their personal welfare [11]. Social media marketing programs integrate four components of the “marketing mix” (or 4 P's): (1) product—the bundle of benefits that derive from the behavior; (2) price—reducing the cost of the behavior; (3) place—the location, time, and setting in which the behavior occurs; and (4) promotion—informing and persuading individuals of the net benefit of the behavior [12]. Social marketing programs go beyond education and attempt to increase the attractiveness of the behaviors and reduce the barriers to behavior adoption [12].

It has been proposed that to promote behavior change, education programs should be based on at-risk populations' beliefs, emotions, and perceived barriers concerning HIV/AIDS and should be able to address such psychosocial needs through systematic and strategic formative evaluation processes [13–15]. This is especially important in the case of HIV/AIDS, due to HIV-related fear which causes maladaptive reactions such as denial, fatalism, and avoidance [15, 16]. Such work, assessing young Latino immigrant MSM attitudes and beliefs about HIV testing, has been conducted by the authors of the current study [17]; in the context of social media marketing, such formative research is considered audience research. This formative research found that 35% of the Latino MSM had never undergone testing. Moreover, compared to testers, nontesters were more likely not to identify as gay, to have sex with both men and women (MSMW), to have less knowledge about HIV risks, and to perceive their sexual behaviors as less risky and to deflect HIV-related stigma [17]. Testers tended to be men who self-identify as being gay. Both groups believed that fear of a positive result was the main barrier to testing, and this belief superseded all others. Both testers and nontesters also reported negative attitudes from family members towards HIV testing and believed that the simple act of seeking testing would be interpreted by family members as a confirmation of being gay and/or of being promiscuous. In addition, both groups believed that having Latino staff at HIV testing sites hinders confidentiality and both groups also expressed concerns about the financial cost of HIV testing.

The objective of the present study is to apply the data from this formative research to develop HIV prevention messages to promote timely HIV testing and then to translate these messages into Public Service Announcements (PSAs) as part of a social media marketing campaign that will target young Latino MSM Spanish speakers. Although the target audience will be MSM who do not identify as gay, the messaging will use language that does not alienate MSM who do identify as gay. Such an approach may allow both groups to benefit from the developed messages for the social media marketing campaign. In summary, this paper (a) describes the development of HIV prevention messages through the integration of previously collected formative data; (b) describes the process of translating these messages into PSAs, including the application of a marketing strategy; (c) describes testing the PSAs within the Latino MSM community; and (c) determines a set of important factors to consider when developing HIV prevention messages for young Latino MSM who do not identify as gay. The ultimate goal is to use developed PSAs for a future social marketing campaign to promote HIV testing among young Latino immigrant MSM.

To date, no social media marketing programs have targeted young Latino immigrant MSM Spanish speakers (of age 18–25) who do not identify as being gay with HIV prevention messages in the USA. A recent Cochrane analysis of existing social media marketing programs that target MSM in developed countries identified only three such programs [18]. However, none of the aforementioned studies were a randomized control trial. The authors recommend that future social media marketing campaigns use more rigorous designs in evaluating social media marketing interventions, measure their long-term impact, and identify intervention components that are most effective in reaching the target population and changing behaviors. The CDC has recently created a campaign that promotes HIV testing (http://www.cdc.gov/features/REASONS-RAZONES/) that reminds Latino gay and bisexual men that there are many reasons for getting an HIV test and that everyone can play a role in stopping the spread of HIV; this campaign's outcomes are currently under evaluation.

## 2. Methods

This project was developed through collaboration between University of WA researchers and Entre Hermanos, a community-based organization serving the Latino lesbian, gay, bisexual, and transgender (LGBT) community in Seattle, WA. We used a community-based participatory research (CBPR) approach [19] in conducting this research; this involved the development of a genuine partnership between the University of WA researchers and Entre Hermanos, capacity building of community members in research, applying findings to benefit all partners, and long-term partnership commitments. Building from this CBPR approach, the researchers developed a campaign that was executed by Activate Brands, based in Denver, Colorado. We now describe how HIV prevention messages were developed using the preliminary formative research, describe the process of testing the messages within the Latino MSM community, and describe important factors to consider when developing HIV prevention messages for young Latino MSM.

### 2.1. Phase One: Development of HIV Prevention Messages through the Integration of Previously Collected Formative Data

**Figure 1:**
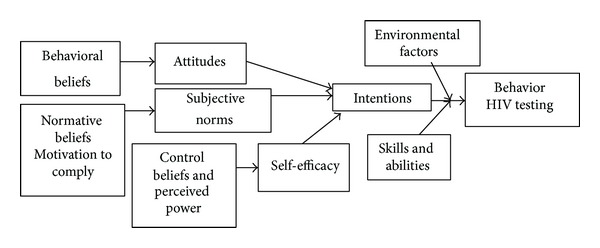
Conceptual model of constructs influencing intentions to undergo HIV testing (adapted from the integrated model of behavior; [20–25]).

Using previously collected formative data [17], a storyboard script was developed to promote HIV testing. We used the integrated model of behavior (IMB; Figure 1); [20–25] as a framework in developing HIV prevention messages for the storyboard script that address young Latino MSM Spanish speakers attitudes, beliefs, norms, and self-efficacy towards HIV testing; the messages developed reframed negative attitudes and beliefs with positive ones. The ultimate goal of the developed messages was to reduce barriers to HIV testing.

The formative research [17] identified important areas to address in the HIV prevention messages. The messages were developed for young Latino immigrant MSM who do not identify as gay (i.e., market segment). Important areas to address were these young men's beliefs and attitudes about HIV risk, perceptions of sexual risk behaviors (i.e., tended to believe that occasional sex with men may not be risky because their sexual partners looked healthy), and HIV-related stigma (i.e., belief that HIV testing is only for promiscuous men or gay men) as well as their self-efficacy towards HIV testing (i.e., fear of undergoing testing because results might be positive, fear of going to an HIV testing site alone without any social support, and fear that their family members might think that they are gay or promiscuous for seeking testing).

The newly developed messages were focused to promote HIV testing.  Table 1 describes the messages used in storyboard script, the rationale for reframing negatives attitudes, beliefs, norms, and self-efficacy with positive ones, and the IMB domain addressed. We systematically countered each of the most prevalent negative perceptions regarding HIV testing. The benefits of timely HIV testing (i.e., individuals are better off knowing their status since they may benefit from treatment) were highlighted. To counter the perception that it is best to defer HIV testing due to the fear of testing positive, we focused on the fact that the majority of Latino MSM who undergo HIV testing will test negative. To counter the perception that a positive result means imminent death, we reframed this belief by positioning HIV as a chronic disease for which treatment exists and with which a person could potentially live a normal lifespan. To counter the denial of risk for HIV, we focused on the fact that MSM sometimes have unprotected sex and therefore, in fact, are at risk. To counter issues of self-efficacy (i.e., men fearing being judged by family members simply for seeking testing) we countered that the men's health takes priority over the concerns of other people; to further enhance feelings of self-efficacy, we promoted getting social support from a trusted friend who could accompany them to HIV testing site. To counter stigma related to the perception that HIV testing is only for MSM, we emphasized that the CDC has recommended HIV testing for all people, including men and women [26].

#### 2.1.1. Storyboard Script Testing with Focus Groups

The storyboard script was tested with ten focus groups (*N* = 61). This approach allowed participants to have discussions about the HIV prevention messages with each other with the goal of yielding information about MSM community norms with regard to HIV testing. Latino MSM were recruited from Entre Hermanos and through flyers posted at community sites. Latino MSM community members were hired and trained to recruit and facilitate focus groups and assist with the interpretation of the qualitative data obtained. Facilitators for each group all had previous experience working with Latino MSM. Written informed consent was obtained from all participants. Focus groups lasted 90 minutes in length. Each focus group was audiotaped and transcribed, and all identifying information was removed from the final transcript. Each focus group participant was paid $25 for their participation. Entre Hermanos permitted project staff to conduct the focus groups with clients of their organization on site, in a private room. The University of WA Institutional Review Board approved this study.

Eligible individuals were biologically male, of Hispanic/Latino descent, between the ages of 18 and 40 years (with an average age of 25), Spanish speaking (monolingual), and reporting a history of having sex with men in the past 12 months. Those with obvious mental or cognitive impairments or under the influence of drugs or alcohol at the time of screening were excluded from study participation.

We used a grounded theory approach with open coding to generate reactions to messages according to participant's experiences and perspectives [27]. We used an iterative process of questioning, analysis, and verification until saturation was reached. A process of comparison between focus group responses was used to consolidate dominant themes and data collection ceased when “saturation was reached” (themes being repeated in successive focus group comments). After the 10 focus groups, the participants no longer had any new information to add to enhance the messages and all participants considered the final messages developed to be acceptable.

#### 2.1.2. Selection of a Communications/Marketing Partner

At the end of phase one, plans were made to select a marketing firm to assist in the translation of developed HIV prevention messages into public service announcements (PSAs) for a media campaign to promote HIV testing. A search for local and national marketing firms with experience working with the Latino Spanish speaking populations was undertaken. Three firms were identified and their experience working with Latinos and proposed budgets were evaluated. Activate Communications, a firm based in Denver, Colorado, was selected.

### 2.2. Phase Two: Translation of HIV Prevention Messages into Public Service Announcements (PSAs)

**Figure 2:**
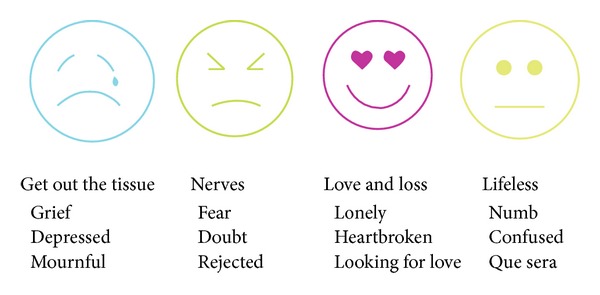
Activate Mood Marketing Chart—Project Pepe.

Activate focuses on interactive campaigns that help organizations formulate and convey messages that explain the purpose behind their brands. Using their proprietary “mood marketing tool” (Figure 2) they create messages that produce positive change in individuals as well as the community in which they reside.

Activate leveraged insights gathered from the previously collected formative data [17] in developing concepts for the campaign. The formative data provided a format for Activate to tap into the mood sentiment of young Latino MSM who may not identify as being gay. The insights gathered and the mood sentiment of these men led to the development of a character that evokes the same emotion they are experiencing towards their identity.

The character “Pepe” formed from the Mood Marketing tool findings, social media outreach, and focus group testing; all of these identified key aspects based on mood that addressed why Latino immigrant MSM do not seek HIV testing in a timely manner. The model for the Pepe campaign is for Latino MSM to view Pepe as a close friend and confidant who will help them receive access to confidential HIV testing and information on safe sex practices. Pepe was created to be a fictional character on purpose to enhance confidentiality. The ultimate goal when this campaign is launched is for the community to see Pepe as an advocate who can provide positive support in a nonjudgmental environment that will encourage testing and prevention. Pepe promotes hope for better quality of life using culturally competent information in Spanish. The focus of the campaign is to increase the number of Latino MSM who get tested for HIV. Ultimately, our goal is to connect the target male consumers with the emotions/moods generated by these messages; we plan to present such moods through social and mobile media to increase participation and interest among young Latino MSM.

Using the formative research insights, multiple PSAs were created that focused on four central themes: community, empowerment, private life, and humor. The intent of these developed messages was to help reduce cultural barriers to testing and to encourage safe sex practices. The formative research thus was used as a platform to create a culturally tailored HIV awareness campaign that the target group could relate to. Three PSAs had a male voice and one had a female voice.

The resulting PSAs incorporated elements of each of the marketing mix components (the 4 P's—product, price, place, and promotion) to promote HIV testing. The formative research provided a clear understanding of young Latino men's fears, concerns, and moods with regard to HIV testing. We incorporated a marketing mix of the 4 P's to reduce the barriers (e.g., price) to HIV testing [28, 29]; Table 2 describes the marketing approach. The main barriers previously identified included fear of testing HIV-positive and associated stigma, lack of perceived benefit, cultural factors, confidentiality concerns, and financial barriers [17]. Thus, the developed messages sought to promote HIV testing by focusing on reducing the barriers and promoting the benefits of testing (Table 2). The overall intervention (i.e., the* product*) is positioned as an outreach program that offers HIV testing in accepting environment andprovides connections to health and social services with an intent to keep testing as anonymous as possible (i.e.,* place*). This overall message was then promoted through the use of peer models in the PSAs themselves.

The peer model promotes testing at multiple HIV testing sites (i.e., place), including medical and nonmedical sites and those within and outside of Latino communities. This approach will allow men to choose a site that meets their needs (i.e., this will reduce fears with regard to confidentiality). In addition, some MSM fear being judged by family/friends simply for seeking HIV testing (i.e., they fear being labeled as promiscuous). These concerns are addressed by focusing on the importance of MSM's health over the concerns of other people. Since the Latino men expressed financial concerns about HIV testing, the developed messages promote free HIV testing at HIV testing sites.

### 2.3. Phase Three: PSA Evaluation

To test the developed PSAs, we conducted four focus groups (*n* = 15) with Latino MSM from September 1, 2013, to November 30, 2013. We specifically targeted young MSM, of age 18–25, for PSA testing. We again used an iterative process of questioning, analysis, and verification until saturation was reached. A process of comparison between focus group responses was used to consolidate dominant themes, and data collection ceased when “saturation was reached” (themes being repeated in successive focus group comments). Focus groups addressed PSA content, theme, and quality of voice/delivery. The focus groups also assessed other campaign elements including photos, emoticons, logos, the call to action, website colors, and Pepe's identity and name. All focus groups were digitally recorded, transcribed verbatim, and imported into ATLAS.ti [30]. All data were deidentified to protect confidentiality. Eligibility criteria, recruitment process, and data analysis mirrored those of the initial focus groups, outlined above, the only exception being that the age group for the PSA testing was 18–25 years.

To assess the content of the PSAs, we assessed message comprehension, perceived impact of messaging, and perceived impact on attitude strength. In addition, evaluation questions measured pre- and post-quantified outcomes, including using a 10-point scale for intentions to undergo HIV testing in the next 3 months and IMB domain assessments, including attitudes, beliefs, norms, and self-efficacy towards HIV testing (see Supplementary Material available online at http://dx.doi.org/10.1155/2014/353092).

### 2.4. Phase Four: Dissemination of HIV Prevention Messages

The PSAs were created for a mass media campaign that will involve radio PSAs, a campaign website (http://tuamigopepe.com/), social media outreach and a campaign awareness, and reminder system using mobile technology and print materials. The PSAs are scheduled for airing between January 15, 2014, and April 15, 2014. The PSAs are also incorporated into campaign website. Evaluation of the media campaign is forthcoming.

## 3. Results 

### 3.1. Participant Characteristics

A total of 66 Latino immigrant men participated in the two sets of focus groups. Both groups were comparable in terms of being mainly of Mexican descent (over 75%) and the rest from another Latin American country. For phase one focus groups, the age ranged from 18 to 40 years with a media age of 25 years and over 60% of the men reported a history of HIV testing; for phase three focus groups, the age ranged from 18 to 25 years and 80% had never been tested for HIV and, among the 20% who had been tested, the testing had taken place more than 12 months ago. All participants were monolingual Spanish speakers. Most participants had less than a high school education, with annual incomes below $20,000. Most of the participants had resided in the USA for less than 5 years and had less than a high school education. Although the majority of MSM (over 70%) self-identified as gay, several explained that their self-identification varies depending on the type of group that they are with (i.e., they may identify as gay when in the presence of other gay friends but not when in the presence of family members). All participants were residents of King County, WA.

### 3.2. Final PSAs

The final PSAS developed were in Spanish language (translated into English below). The PSAs focused on four central themes: community, empowerment, private life, and humor. Out of 8 PSAs developed (using two voices for each one), four were selected as being the best ones by the focus groups. The finalized PSAs are presented below.

*Community. Hi, my name is Julia, and I am dedicated to provide care and support to the men in the community of King County who are seeking a private, very rapid, and confidential option to take a free HIV test. It is for them that we have created Pepe, through which by just texting CONOCEAPEPE to 99000, you will receive in the privacy of your phone the information you need to take that important step. Come on, we are waiting for you—and remember that for Latino young men, 90% of the time their results are good news. And you, have you met Pepe? Find out more at TuAmigoPepe.com.*

*Hola, mi nombre es Julia, y me dedico a brindarles atención y apoyo a los hombres de la comunidad de King County que buscan una opción privada, muy rápida y confidencial para hacerse una prueba gratuita del VIH. Es para ellos que hemos creado a Pepe con el cual, a través de tan sólo enviar el mensaje CONOCEAPEPE al 99000, recibirás en la privacidad de tu teléfono, la información que necesitas para dar ese importante pasó. Acércate, te esperamos—y recuerda que para los hombres latinos jóvenes, el 90% de las veces sus VIH resultados son buenas noticias. Y tú, *¿*ya conociste a Pepe? Averigua más en TuAmigoPepe.com.*

The preliminary research identified that Latino men wanted “to be invited” to come in for testing and wanted information about sites that offered free HIV testing. Therefore, we developed the messages to be inviting and each was delivered from a friendly community provider of HIV testing services. In previous focus groups, the men had said:

*The peer model needs to invite others to undergo testing…it must be done with love and knowledge.*

*People need to be invited for testing without instilling them with fear and without the use of strange words…I've heard programs on the radio that promote HIV testing and they say to undergo testing before it's too late but instead, they should say, undergo testing and you might live a better life.*

Due to the preliminary research indicating that the main barrier to HIV testing is being fear of a positive result, we considered it important to emphasize to participants that in the majority of cases (i.e., 90%) the test result will be negative, since this is consistent with the current epidemiology of HIV among Latinos [31]. When this message was tested with focus groups, the participants particularly liked the statement, “…over 90% of young Latinos who test for HIV will test negative.” Participants also liked the recommendation to go to the campaign website for additional information and the fact that such a task could be done anonymously.

*Empowerment. I consider myself a man sure of what I am, what I feel and want. I take my health and well-being seriously. That's why I like talking about the importance of getting tested for HIV. Thanks to Pepe, I took the test with total privacy, confidentiality, and for free. I got the results in only 20 minutes and with service in Spanish. Now I feel even more confident. And you can too. Just think that to have fear does not help…but to have courage does. Besides, for Latino young men, 90% of the time results are good news. And you, have you met Pepe? Text CONOCEAPEPE (MEETPEPE) to 99000 and you too, get tested.*

*Me considero un hombre seguro de lo que soy, de lo que siento y quiero. Yo me tomo en serio mi salud y bienestar. Por eso, me gusta hablar de la importancia de hacerse la prueba del VIH. Gracias a Pepe, me hice la prueba con total privacidad, confidencialidad y de manera gratuita. Tuve los resultados en sólo 20 minutos, y además me atendieron en español. Ahora me siento todavía más seguro. Y tú también puedes. Sólo piensa que tener miedo, no ayuda*…*pero tener valor, sí.*

*Además, para los hombres latinos jóvenes, el 90% de las veces los VIH resultados son buenas noticias. *

*Y tú, *¿*ya conociste a Pepe? Envía el mensaje CONOCEAPEPE al 99000 y tú también hazte la prueba.*

The focus group participants considered it important to emphasize health and well-being and to promote HIV testing within such a context. In addition, confidentiality is extremely important to Latino men and thus the men recommended that messages should increase awareness about multiple places for HIV testing that offered confidential services.

The original script contained the statement, “I am proud to be a gay Latino man.” Some men objected to that statement, noting that not all Latino MSM self-identify as gay—and among those who do, not all are “proud” to be gay. They therefore felt that some MSM would not relate to this type of message. This stimulated a lively discussion about the statement “I am proud to be gay” among young Latino MSM. Most men felt that being gay is no longer a negative stereotype, as we now live in a more open-minded society:

*“Someone may not be gay but they have friends who are gay…they may go to gay bars together, they live with gays.”*

Participants felt that, previously, more Latino men hid the fact that they were gay, but now many more social networks exist for gay men, and it was easier for gay men to find other gay men like themselves. The men said that nowadays there is increasing recognition about the wide spectrum of self-identification among MSM, with some identifying as gay and others as bisexual, transgender, or heterosexual. In general, the men perceived that young people are growing more comfortable with their sexuality. However, this does not negate the fact that stigma remains associated with being an MSM and the men especially perceive a lack of acceptance from family members:

*Perhaps, some young Latino MSM want to be like the messenger, open about being gay, but they cannot because of what their family might say.*

*We [gays] perceive that family will reject us if we come out, and even though we may identify with being gay, we still feel lost and feel that we do not belong anywhere. The issue is not just coming out of the closet but how you identify yourself. However, due to perceptions about how family will treat us if they find out, we prefer to not even investigate the sexual identify inside ourselves.*

The men also thought that if a young man is currently “living in the closet,” the message about admitting that he is gay might make him feel uncomfortable. The men expressed a concern that the messenger sounded overly confident in being gay and that men who did not feel secure about their sexual orientation may not identify with him and therefore may not absorb the rest of the message and may not text into the campaign website to obtain more information.

Some participants felt that even if some young Latino MSM identify as gay, they may still choose to stay quiet about this in public. According to participants, many men who identify as gay feel as if they live in two separate worlds: one with other gay men, in which they feel free, and another with family, in which they have to hide a portion of their identity. A minority of men expressed the opinion that some young Latino MSM who identify as gay may feel supported by a message asserting “I am gay” because this message indicates that he knows who he is, is comfortable with his identity, and therefore got tested for HIV.

In general, the men wanted the message to be more personable and for the tone to sound less “pushy” (i.e., to not tell them how to self-identify or how to feel). The men liked hearing about the importance of being strong and overcoming fear of testing. In the final iteration of the message, the statement about being proud and being gay was removed based on participant feedback. This was done because our goal with the messages is to target MSM who may not identify as gay while still resonating with MSM who do not identify as gay.

*Private Life. I am one of those who keep things to myself. In the end, not everyone understands what I prefer to do with my life. But there is something that I find important, and that is to know what happens with my body and my health. So I was glad to know that I could get tested for HIV with complete confidentiality, for free, and with service in Spanish. Thanks to Pepe, I visited the X clinic and in only 20 minutes I had the results in my hands. And here is where the results will stay. You need to be encouraged also—besides, as young male Latinos we have something in our favor and that is that 90% of the time, our results is good news. Not everyone has to but you should know it. And you, have you met Pepe? Text right now CONOCEAPEPE (MEETPEPE) to 99000.*

*Yo soy de esos que se guardan las cosas. Al final, no todo el mundo entiende lo que prefiero hacer con mi vida. Pero hay algo que sí encuentro importante, y es saber lo que sucede con mi cuerpo y mi salud. Por eso, me alegró saber que podía hacerme la prueba del VIH con total confidencialidad, de manera gratuita, y con atención en español. Gracias a Pepe, visité la clínica X y en sólo 20 minutos tuve los resultados en mis manos. Y aquí se quedan. Anímate tú también—además, los hombres latinos jóvenes tenemos algo a nuestro favor y es que el 90% de las veces, nuestros resultados son buenas noticias. No todos tienen que saberlo pero tú sí debes saberlo. Y tú, *¿*ya conociste a Pepe? Envía ahora mismo el mensaje CONOCEAPEPE al 99000.*

Most participants stated that they preferred to keep their sexual orientation to themselves. Several reported that their friends did not know that they were having sex with other men and that they would not understand their behavior. Many young Latino men expressed how difficult it was to “come out” to friends and family about being gay and this message was developed to reflect that.

When we tested this message with focus group, the participants liked the story and felt that the message had authenticity. The men especially liked that the messenger did not have a perfect attitude (i.e., his voice sounded shaky and timid and slightly depressed in the first half of the message but then as he asserts that he cares about his health, his tone changes to be more lively and confident) and that he was not perfect in general, making him “more real” and easier for other young Latino MSM to relate to him. Participants felt that Latino men will identify with this message. They liked the type of words used and felt that the messenger's shaky tone was particularly authentic in its portrayal of a young Latino man just “coming out”:

*[This message] makes you think, oh, I am not the only one who feels this way…there are other people who have the same questions that I have [*i.e.,* about their sexuality] or who feel the same way as I do [*i.e.,* depressed].*

The participants felt that this authenticity may influence other Latino men to identify with the message. Furthermore, the men appreciated that the messenger cared about his health and liked his message about where to go to get tested and the importance of knowing one's HIV status

Participants were asked, “What could cause you to be willing to follow the advice in this message?” They responded that if the message focused more on respecting one's body they might be more inclined to follow the messenger's advice. The men recommended removing the words from the original message, “nobody should care about what I do” (stated in reference to sexual behaviors), because they felt such words have negative implications and are not consistent with the outlook of young Latino MSM (the participants said that young Latino MSM do care about what others think and that they do need social support). The men recommended that the messages should focus more on that fact that the messenger underwent testing because his health matters to him. The men also wanted the message to promote the idea that those who undergo HIV testing are strong and felt that the message should emphasize that the messenger does not feel ashamed for undergoing HIV testing.

One man commented on the timid and shaky tone of the message, stating that not all Latino men are timid and wondering if this would promote untrue stereotypes about Latino MSM. However, the majority of men disagreed and said that young MSM are all in different phases of self-identity with regard to their sexual orientation and therefore it would be a good idea to have many different types of messages to reach a broader audience.

*Humor. In the closet, out of the closet, who cares about the closet…Text CONOCEAPEPE to 99000 right now and discover that taking a free HIV test is easier and more private than you think. Plus, you get the results in only 20 minutes. And speaking of results, 90% of the time they are good news for Latino young men. Thank you Pepe…it's good you came into my life, friend. Find out more at TuAmigoPepe.com.*

*Pssss…have you met Pepe? Text TUAMIGOPEPE to 99000 right now and discover that taking a free HIV test is easier and more private than you think. Plus, you get the results in only 20 minutes. And speaking of results, most of the time they are good news for Latino young men. Thanks, Pepe…it's good you came into my life, friend. Find out more at TuAmigoPepe.com. Have you met Pepe? Text CONOCEAPEPE (MEETPEPE) to 99000 right now.*

*Dentro del clóset, fuera del clóset, a quién le importa el clóset…Envía ahora mismo el mensaje CONOCEAPEPE al 99000 y descubre que hacerte una prueba gratuita del VIH es más fácil y privado de lo que te imaginas. Gracias, Pepe…qué bueno que llegaste a mi vida, amigo.. Averigua más en TuAmigoPepe.com.*

*Pssss…*¿*ya conociste a Pepe? Envía ahora mismo el mensaje TUAMIGOPEPE al 99000 y descubre que hacerte una prueba gratuita del VIH es más fácil y privado de lo que te imaginas. Además, obtienes los resultados en sólo 20 minutos. Y hablando de resultados…la mayoría de las veces son buenas noticias para los hombres latinos*

*jóvenes. Gracias, Pepe…qué bueno que llegaste a mi vida, amigo.. Averigua más enTuAmigoPepe.com.*

**¿*Ya conociste a Pepe? Envía ahora mismo el mensaje CONOCEAPEPE al 99000.*

Many participants had previously expressed that they were still living “in the closet” and that their family members and friends did not know that they were having sex with men. Therefore this message was developed to be inclusive of all men, whether they are “in the closet” or “out of the closet.”

When we tested this message with focus groups, the men felt positive about this message and found it easy to relate to, funny, friendly, and effective. Participants perceived that many young Latino MSM would identify with this message. The men considered this message inclusive of all men, regardless of self-identification of sexual orientation:

*“It does not matter at what phase you are in, in terms of sexuality and self-identification, the point is to be tested and to meet Pepe.”*

The men liked how the message was inclusive of persons “in the closet” or “out of the closet” and felt that message targeted all MSM, regardless of whether they identified as being gay. The men believed that such an inclusive message would have a great impact on young Latino MSM.

While the majority of participants felt that all listeners to this message would know what “closet” refers to, one man wondered if young Latino MSM would understand the use of this word:

*Young men are curious and are trying to figure out what it means to be gay; but if a young man is confused and is not at a point in their life where they want to find out about themselves, they may not know what the term closet refers to. When you are part of the gay community, after several years, you begin to understand this term (*i.e.,* closet). However, when you are not part of the community, it may be hard to understand this term. *

In general, participants liked the tone of this message and the way the story was structured. The men stated that this message was one that they wanted to keep hearing:

*“This young man is part of our circle. He sounds like a friend who is talking with you. He speaks the way we would speak.”*

The original message included words like “On top of the closet…under the closet” and the men recommended removing those words because they felt that the message would be better stated in a simpler form.

All of these final PSAs were deemed effective at promoting HIV testing by the focus groups. The majority of participants reported having intentions towards HIV testing in the next 3 months after hearing the PSAs. Using quantitative surveys to assess the domains from the IBM (i.e., beliefs, attitudes, norms, and self-efficacy), a general trend was observed among the focus group participants towards more positive attitudes, beliefs, norms, and self-efficacy. 

#### 3.2.1. Campaign Components

*Voices*

*(1) Female and Male Voices*. We tested scripts with female and male voices and the participants selected the ones they liked best. One message with a female voice and three messages with male voices were selected out of 8 messages tested.

The female voice of Julia in the “Community” PSA was selected because her voice sounded natural and friendly. Her voice reminded participants of a community counselor. The initial groups considered one female voice as being “too sexy” and recommended changing it; this voice was removed from the messages.

*(2) Empowerment PSA Voice*. Focus group participants also described concerns with the voice in the “Empowerment” PSA. They liked that his voice sounded decisive but felt that it needed to express more enthusiasm and sound more youthful in order to call attention from young Latino men about his message. Their recommendations were followed in the final PSA.

*(3) Private Life PSA Voice*. In the “Private Life” PSA, the men felt that the timidity and shakiness of the messenger's voice conferred authenticity to his message.

*(4) Humor PSA Voice*. The men liked the enthusiasm and the youthfulness of the messenger's voice in the “Humor” PSA.

*Images. *The men were shown nine photographs of young Latino men. They were asked to pick the ones that they thought would best promote HIV testing. A photograph of two men hugging was the most preferred by the largest proportion of participants. This photo represented warmth, caring, social support, and trust. Other images that the men liked included a photo of a young construction worker because it depicted an ordinary young working-class man and his face was easily recognized as Latino. Some men felt that the construction worker's face might invite a broader audience (i.e., not just those who identify as being gay, as some participants believed that the photo of two men together implied that self-identification):

*Let's say you are not out of the closet or you do not want to identify as being gay and if I put this photo of two men, together, in front of you, then you will not want to know anything else; on the other hand, the photo of the construction worker could be of a man who is gay or straight…he could be in or out of the closet…he might have children.***

*Emoticons. *Various emoticons were also tested with the focus groups. The preferred emoticon depicted a cell phone image with a smiling face. None of the participants liked the emoticons with mustaches and the group recommended removing those; the men said that they could not identify with the mustache because it made the emoticon look older than 25 years.

*Logos. *Various logos were tested with focus groups. The men recommended that if the emoticon smiles then the campaign logo should not smile.

*Pepe's Image and Name*. The majority of participants supported the idea of including Pepe as a fictitious character. Multiple face forms were tested and the participants chose the one that looked more like a real man rather than an image that was just made up. The participants like the name Pepe because it is a traditional Latino name and easy to remember.

The name Pepe is a play on words on the letter “P” (in Spanish the letter “p” is pronounced “Pe” and two P's together would be pronounced Pepe). We are using the name Pepe as a code name for two Spanish words that begin with the letter “P”, privacy and prevention (privacidad y prevencion).

*Call to Action.* The men approved of the call to action,“Text CONOCEAPEPE” (Spanish for “Meet Pepe”).

*Colors. *Participants were shown photographs with various background colors (potential formats for campaign poster and website). The most popular formats were those with red, blue, and green in the background. One man strongly objected to the color pink, believing that such a color implied that one identified with being gay, and said that he would be embarrassed to open a web page with such color. The preferred color was blue. Most men approved of the use of the color red and felt that this color is appreciated among Latino culture because if reflects life and passion, but some men argued against its use in an HIV campaign, stating that although they like the color red, they did not think it was appropriate for an HIV-related campaign (i.e., it might make some think of danger or blood).

## 4. Discussion/Conclusion

We developed HIV prevention messages that resonated strongly with young Latino MSM. Our approach was grounded in the integrated model of behavior [20–25] and capitalized on a customized program that uses marketing principles to reduce the barriers to HIV testing [28, 29]. The HIV prevention messages developed (i.e., PSAs), targeting Latino MSM key attitudes, beliefs, and norms toward HIV testing, have the potential to influence these men's intentions and HIV testing behavior. To promote HIV testing, messages focused on addressing the barriers (i.e., price) to testing by reducing fear and stigma and addressing cultural factors. In collaboration with Activate, four PSAs were developed that were culturally tailored for Latino immigrant MSM Spanish speakers and these focused on four central themes (i.e., community, empowerment, private life, and humor) to reduce fear and stigma related to HIV testing.

The preliminary formative research identified the costs associated with HIV testing (price); identified the most salient benefits of testing from the audience's perspective and used these benefits in the development of marketing offers (product); and identified channels for product delivery (place). Marketing segmentation is a marketing strategy that involves dividing a broad target market into subsets of consumers who have common needs and priorities. By using attributes of the target audience such as demographics, behaviors, beliefs/attitudes, segments (smaller, more homogeneous, and meaningful subgroups) were created (i.e., young Latino MSM who do not identify as gay); such an approach will influence the final social marketing program design and outcome. Finally, the product will be delivered (promotion) to these segments through selected channels (e.g., mass media, including radio PSAs, campaign website, and social media outreach).

The feedback from the focus groups on the development of these HIV prevention messages was quite positive and thus provides promising preliminary data for the development of a mass media campaign to target Latino MSM with HIV prevention messages, including those focused on HIV testing.

This research represents an initial step in the development of theoretically based HIV prevention messages for Latino MSM that may be used in social marketing campaigns to promote HIV testing. However, as with most research studies, there are limitations in the current study that need to be discussed. First, the participants in this study tended to be monolingual Spanish speakers of Mexican descent and the majority had lived in the USA for less than 5 years; thus, the findings presented here are from a group that appears to have low levels of acculturation to US culture. Second while the majority of the sample identified as being gay, many men explained that such self-identification varies by the type of group that they are with, with some only identifying as gay when in the presence of other gay friends but not when in the presence of family members. Thus, since the sample was recruited from Entre Hermanos, a community-based agency serving the needs of the Latino LGBT community, it is highly possible that many of the men who self-identified as being gay may not do so at other places. Additionally, it is also possible that the some Latino MSM who do not identfy as gay may not be comfortable seeking services at Entre Hermanos. Thus, the type of men who are represented here may be a group that is somewhere in the middle of the continuum of self-identification with being gay. Current studies describe various methodologies used in recruiting MSM who do not identify as gay, including recruitment from community agencies, as done in our study, and snowball sampling [32–34].

Previous studies report that Latino immigrant MSM are less likely to identify as being gay compared to Latino US-born MSM; in addition MSM immigrants of Mexican descent are less likely to identify as gay compared to MSM from other Latino ancestries [35]. Thus, future campaigns that target Latino immigrant MSM of Mexican descent need to take this into consideration and develop HIV prevention messages that target MSM who do not identify as gay; considering that self-identification may be a continuum and such self-identification may change over time, it would be important for such campaigns to not alienate MSM who do not identify as gay.

Our findings have implications for future HIV prevention interventions targeting young Latino MSM who do not identify as gay. The next step of this research will involve the incorporation of the developed PSAs into a mass media campaign to promote HIV testing. The campaign will include radio PSAs, a campaign website (http://tuamigopepe.com/), social media outreach, campaign awareness, and reminder system using mobile technology and print materials. Once these PSAs are tested in a pilot social marketing campaign and shown to be effective at promoting HIV testing, they may ultimately be used in radio, television, and social media websites to promote HIV testing among young Latino MSM.

## Supplementary Material

Integrated IMB constructs measure attitudes, beliefs, norms and self-efficacy associated with HIV testing. These constructs assess whether attitudes and beliefs are positive or negative. For each construct, a scale score is computed by taking the mean of the items measuring the construct.

## Figures and Tables

**Table 1:** Reframing negative HIV testing attitudes, beliefs, and norms with positive ones.

Messages in storyboard script	Rationale for reframing negative beliefs, attitudes, and norms with positive ones.	Integrated model of behavior domain addressed
Testing is beneficial for me. My life matters to me.	Young MSM need help in prioritizing their health over the concerns of other people when contemplating HIV testing.	Positive belief

I tested for HIV and my results are negative.	This is the likely outcome for most young Latino MSM testing for HIV and knowing this may deter them from further delaying testing.	Positive belief about behavior (testing)

HIV is a chronic illness that can be treated with medications.	Many MSM are fatalistic and consider that a positive result means that they are going to die very soon. This statement contradicts that common belief.	Countering negative beliefs

I did not think that I was at risk for HIV and therefore did not think that there was a need for testing; however, there were times when I had not used protection (condoms).	Many Latino MSM are in denial about being at risk for HIV; however, they need to understand that if they did have unprotected sex, then they do need to get HIV screening.	Countering negative beliefs

What will my family say? What will my friends say? My health is on the line and I need to know my status.	MSM need to know that they are better off knowing their HIV status. They fear that family members and friends will consider them promiscuous for seeking HIV testing.	Self-efficacy

My friend, Carlos, helped me out. He accompanied me to the HIV testing site. He calmed me down while I awaited my results.	Men need peer social support when seeking HIV testing (i.e., to reduce stress and fear).	Self-efficacy

HIV testing is available at many places, including medical clinics, emergency room, hospitals, community-based organizations, and HIV testing centers funded by public health.	Latino MSM have confidentiality concerns about Latino staff at HIV testing centers (they fear that staff will spread rumors about them for seeking testing); thus, informing them of the multiple places one can go for testing may increase their likelihood of identifying a place where they may be more comfortable seeking testing. We plan to promote multiple HIV testing sites, including Entre Hermanos, Gay City, and other sites that offer free HIV testing.	Negative beliefs

I test for HIV every 6 months. The CDC recommends that everyone, man or woman, of age 13–64 years, receive an HIV test. Persons who remain at risk, including men who have sex with men, are advised to undergo HIV testing at least yearly.	This statement targets everyone but ends by focusing specifically on young Latino MSM. The MSM recommended that everyone be targeted for HIV testing; they do not want only MSM to be targeted, especially because they are not the only ones at risk.	Normative beliefs

I recommend HIV testing to all of my friends. It is important to protect not just our own health but also that of our community.	Latino MSM tend to express fraternalism.	Positive beliefs

**Table 2:** Application of marketing principles: the 4 P's (product, place, price, and promotion).

Product	The product is the intervention itself which promotes Latino men's sexual health and offers HIV testing in an accepting environment that provides social support and linkages to needed health services

Place	Multiple HIV testing sites are promoted within and outside of Latino communities and these sites include medical and nonmedical sites (i.e., local health care facilities, Gay City, and hospitals); this way MSM may identify HIV testing sites that meet their needs

Price	We used a marketing mix of the 4 P's to reduce the “price” of HIV testing (i.e., reduced the barriers to testing)

Promotion	The peer model promotes the desired behavior (i.e., HIV testing)
