# Supplementary material for: HIV Prevention Messages Targeting Young Latino Immigrant MSM
Source: AIDS Res Treat. 2014 Apr 17;2014:353092. doi: 10.1155/2014/353092 (PMC4016876; doi:10.1155/2014/353092)
Supplement: Supplementary file 1 — Integrated IMB constructs measure attitudes, beliefs, norms and self-efficacy associated with HIV testing. These constructs assess whether attitudes and beliefs are positive or negative. For each construct, a scale score is computed by taking the mean of the items measuring the construct. [file 353092.f1.pdf]

Evaluation of HIV Testing Intentions

A. MC Intention

1. How likely are you to get tested for HIV in the next month?

0	1	2	3	4	5	6	7	8	9	10
**Definitely Won't**										**Definitely Will**

B. Attitude

1. Getting tested for HIV in the next month would be:

		Extremely	Quite	Neither	Quite	Extremely	
		1	2	3	4	5	
a.	***Good Idea***	❑	❑	❑	❑	❑	**Bad Idea**
b.	**Easy**	❑	❑	❑	❑	❑	**Difficult**
c.	**Safe**	❑	❑	❑	❑	❑	**Risky**
d.	**Comforting**	❑	❑	❑	❑	❑	**Stressful**
e.	**Helpful**	❑	❑	❑	❑	❑	**Useless**
f.	**Reassuring**	❑	❑	❑	❑	❑	**Worrying**
g.	**Worthwhile**	❑	❑	❑	❑	❑	**Pointless**
h.	**Smart**	❑	❑	❑	❑	❑	**Dumb**
i.	**Beneficial**	❑	❑	❑	❑	❑	**Harmful**
j.	**Desirable**	❑	❑	❑	❑	❑	**Undesirable**
k.	**Healthy**	❑	❑	❑	❑	❑	**Unhealthy**

C. Beliefs

Next are some statements about getting tested for HIV. Please tell me how strongly you agree or disagree with each statement.

1. If <u>you</u> were to get an HIV test in the next month:

	Strongly Disagree 1	Somewhat Disagree 2	Neither /not sure 3	Somewhat Agree 4	Strongly Agree 5
a. You expect the result to be negative	❑	❑	❑	❑	❑
b. You expect that you can get the test for free	❑	❑	❑	❑	❑
c. You expect you can get the HIV testing results within 20 minutes	❑	❑	❑	❑	❑
d. Will give you the opportunity to practice safe sex	❑	❑	❑	❑	❑
e. The clinic staff may spread rumors about you	❑	❑	❑	❑	❑
f. You expect it to bring you peace of mind	❑	❑	❑	❑	❑
g. You fear getting the HIV test result	❑	❑	❑	❑	❑
h. Other beliefs____________________					

E. Self-Efficacy

1. A variety of things can get in the way of your getting an HIV test. If you want to get an HIV test in the next month, how certain are you that you could?

Certain Certain

I Could Not ______ : ______ : ______ : ______: ______ I Could

extremely quite neither quite extremely

2. If you wanted to get an HIV test, there might be different things that would make it difficult.

For each of the following questions, please tell me how certain you are that you could get an HIV test in the next month under various circumstances.

	Extremely certain I **could not** 1	Quite certain I **could not** 2	Neither /not sure 3	Quite certain I **could** 4	Extremely certain I **could** 5
a. How certain are you that you could get tested for HIV at your local clinic?	❑	❑	❑	❑	❑
b. How certain are you that you could get tested at an HIV testing center	❑	❑	❑	❑	❑
c. How certain are you that you could get tested at home, using a kit?					
d. If you feel fear about your HIV test results, how certain are yo u that you could be tested for HIV?	❑	❑	❑	❑	❑
e. If you have to pay to be tested, how certain are you that you could be tested for HIV?	❑	❑	❑	❑	❑
f. If you are concerned about what other people might say, how certain are you that you could get tested for HIV?	❑	❑	❑	❑	❑

F. Injunctive Norm

Next are questions about some people or things in your life that may or may not encourage you to get an HIV test.

1. How strongly do you agree or disagree that each of the following people or things would encourage you to get an HIV test?

	Strongly Disagree 1	Somewhat Disagree 2	Neither /not sure 3	Somewhat Agree 4	Strongly Agree 5
a. Most people who are important to you	❑	❑	❑	❑	❑
b. People in your community	❑	❑	❑	❑	❑

	Strongly Disagree 1	Somewhat Disagree 2	Neither /not sure 3	Somewhat Agree 4	Strongly Agree 5
c. Your family	❑	❑	❑	❑	❑
d. Your best friend	❑	❑	❑	❑	❑
e. [Add other important referents]	❑	❑	❑	❑	❑
